# Glycine receptor activation promotes pancreatic islet cell proliferation via the PI3K/mTORC1/p70S6K pathway

**DOI:** 10.1172/jci.insight.178754

**Published:** 2025-04-22

**Authors:** Ziyi Zhang, Wenyue W. Ye, Anthony L. Piro, Dian-Shi Wang, Ashley Untereiner, Sulayman A. Lyons, Alpana Bhattacharjee, Ishnoor Singh, Jacqueline L. Beaudry, Beverley A. Orser, Feihan F. Dai, Michael B. Wheeler

**Affiliations:** 1 Department of Endocrinology, Sir Run Run Shaw Hospital, Zhejiang University, Hangzhou, Zhejiang, China.; 2 Department of Physiology and; 3 Department of Nutritional Sciences, Temerty Faculty of Medicine, University of Toronto, Toronto, Ontario, Canada.; 4 Metabolism Research Group, Division of Advanced Diagnostics, Toronto General Hospital Research Institute, Toronto, Ontario, Canada.

**Keywords:** Endocrinology, Beta cells, Diabetes, Islet cells

## Abstract

Glycine and β-alanine activate glycine receptors (GlyRs), with glycine known to enhance insulin secretion from pancreatic islet β cells, primarily through GlyR activation. However, the effects of GlyR activation on β cell proliferation have not been examined. Here, we aim to investigate the potential proliferative effects of glycine and β-alanine on islets. In vitro experiments on mouse and human islets revealed that glycine and β-alanine, via GlyR activation, stimulated the proliferation of β cells and α cells, without affecting insulin or glucagon secretion. Further analysis indicated the involvement of the PI3K/mTORC1/p70S6K signaling pathway in this process. Inhibition of GlyRs and PI3K/mTORC1/p70S6K signaling attenuated proliferative effects of glycine and β-alanine. In vivo and ex vivo studies supported these findings, showing increased β and α cell mass after 12 weeks of oral administration of glycine and β-alanine, with no changes in insulin secretion or glucose homeostasis under normal conditions. However, during an acute insulin resistance induced by insulin receptor antagonist S961, glycine and β-alanine enhanced insulin secretion and reduced blood glucose levels by increasing β cell secretory capacity. These findings demonstrate glycine and β-alanine in vivo and in vitro promote islet cell proliferation via GlyR activation and the PI3K/mTORC1/p70S6K pathway, potentially providing a target to enhance islet capacity.

## Abstract 2

Activation of glycine receptors promotes islet cell proliferation via the PI3K/mTORC1/p70S6K signaling pathways.

## Introduction

Type 2 diabetes (T2D) is characterized by impaired glucose homeostasis caused by insufficient insulin secretion from pancreatic β cells in the face of insulin resistance (1). As a result, methods aimed at early diagnosis of T2D and therapeutic strategies to enhance β cell mass and function have been a major focus of recent research efforts. To this end, glycine supplementation has been shown to increase circulating insulin (2–4), making this nonessential amino acid a possible target for enhancing insulin secretion. It has further been demonstrated that glycine enhances insulin secretion directly from human β cells of T2D and non-T2D donors (5) and that circulating levels of glycine are inversely correlated with T2D risk (6–11), insulin resistance (12–14), impaired glucose tolerance (15, 16), and obesity (14, 17). Taken together, these studies suggest that increased levels of glycine augment β cell function, and low levels of circulating glycine are associated with dysmetabolism and T2D.

In the CNS, glycine acts primarily as an inhibitory neurotransmitter, activating ligand-gated chloride channels, also known as glycine receptors (GlyRs), to generate inhibitory postsynaptic potentials that are important for processing sensory and motor cues (18, 19). Recently, it has been shown that GlyRs, notably α1 subunit–containing GlyRs, are expressed in β cells and that glycine, via GlyRs, stimulates insulin secretion. Mechanistically, it was shown that glycine initiated depolarizing Cl^–^ currents in β cells that enhance action potential firing and Ca^2+^ entry triggering insulin secretion. Thus, in β cells, glycine appears to act as an excitatory neurotransmitter via GlyRs. Besides glycine, GlyRs can also be pharmacologically activated by other amino acids, including β-alanine and taurine (20, 21).

γ-Aminobutyric acid (GABA) type A (GABA_A_) receptors are also permeable to Cl^–^, and their general structure mirrors that of GlyRs. Numerous studies have shown that GABA_A_ receptors are expressed in pancreatic islet cells and that GABA can stimulate both α and β cell proliferation. To this end, we have previously demonstrated that GABA administration increases β cell mass to enhance insulin secretion and augment glucose tolerance in vivo (22). GABA treatment also promotes β cell proliferation via the GABA_A_ receptor through the phosphatidylinositol-3 kinase/ mammalian target of rapamycin complex 1/p70S6K (PI3K/ mTORC1/p70S6K) pathway in vitro (22, 23). Given the fact that GABA_A_ receptors and GlyRs are both ionotropic receptors and that their activation leads to increased Cl^–^ ion conductance, we hypothesize that GlyRs activation may also lead to the proliferation of islet cells. Indeed, glycine has been shown to be associated with cellular proliferation in multiple tissues and cells. Jain and colleagues reported that glycine consumption and the mitochondrial glycine biosynthetic pathway were strongly correlated with proliferation across cancer cells, whereas blocking glycine uptake and biosynthesis led to impaired cell proliferation (24). Lin et al. demonstrated that glycine could enhance satellite cell proliferation through activating mTORC1 (25). Glycine also activates the Akt/mTOR signaling pathway in primary mouse hepatocytes and may play a role in liver regeneration (26). Overall, these findings suggest that glycine can promote cell proliferation, but the effects of GlyR activation on islet cell proliferation still need to be clarified.

Therefore, in the present study, we aimed to examine the effects of glycine and β-alanine, which both activate GlyRs (19), on the islet proliferation and function both in vivo and in vitro, and we explore the potential mechanism underlying those effects.

## Results

### Effects of glycine and β-alanine on islet cell proliferation in primary mouse islets and human islets in vitro.

**Figure 1:**
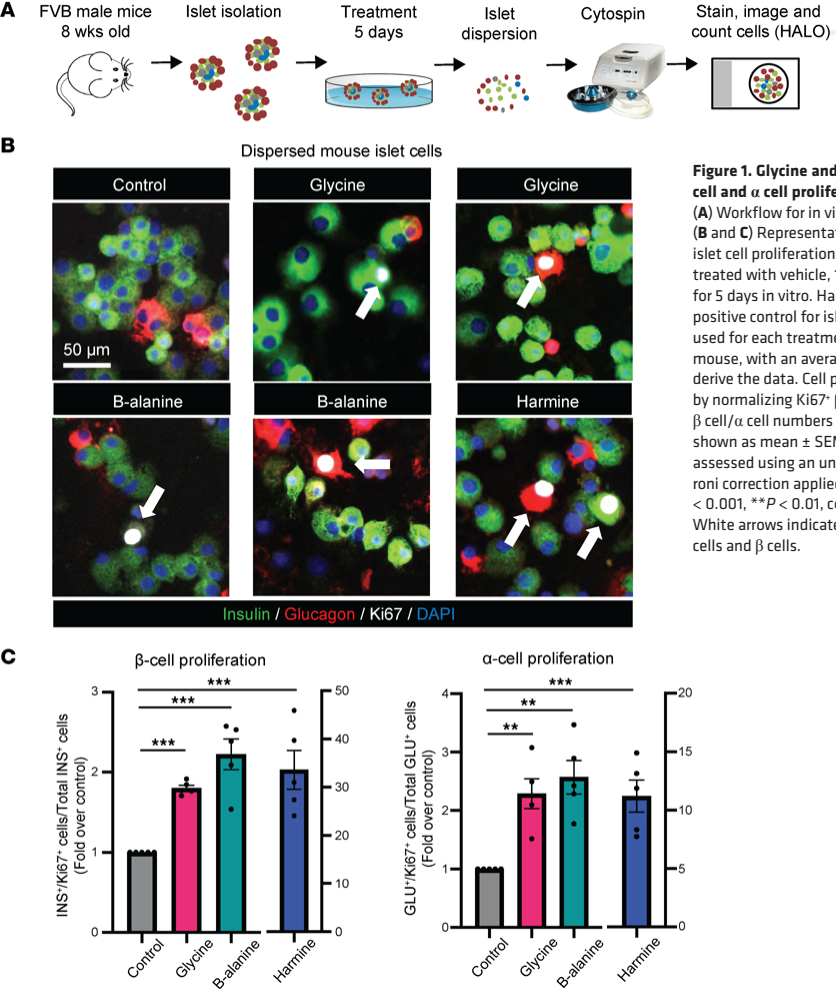
Glycine and β-alanine stimulate both β cell and α cell proliferation in mouse islets in vitro. (**A**) Workflow for in vitro treatment of mouse islets. (**B** and **C**) Representative images of Ki67^+^ cells and islet cell proliferation measurement in mouse islets treated with vehicle, 1 mM glycine, or 1 mM β-alanine for 5 days in vitro. Harmine (10 μM) was used as a positive control for islet cell proliferation (*n* = 5 mice used for each treatment). We used 30 islets per mouse, with an average of 9,761 cells per sample to derive the data. Cell proliferation rate was calculated by normalizing Ki67^+^ β cell/α cell numbers to total β cell/α cell numbers on cytospin slides. Data are shown as mean ± SEM. Statistical significance was assessed using an unpaired *t* test, with Holm-Bonferroni correction applied for multiple comparisons. ***P < 0.001, **P < 0.01, compared with the control group. White arrows indicate Ki67-positive proliferating α cells and β cells.

To investigate whether glycine and β-alanine, which both activate GlyRs, had direct effects on islet cell proliferation, mouse islets were treated with 1 mM glycine or 1 mM β-alanine for 5 days (Figure 1A) in vitro. Since interstitial glycine concentrations are expected to exceed the circulating level (~300 μM), in particular because pancreatic β cells store and release glycine as previously described by Yan-Do et al. (5), we rationalized the use of 1 mM glycine or β-alanine to treat primary mouse and human islets in the present study. Harmine, a DYRK1A inhibitor and mitogenic compound, was used as positive control of islet cell proliferation (27). Ki67 staining was used to evaluate the proliferation of both β cells and α cells in dispersed islets. In mouse islets, glycine treatment significantly increased costaining of insulin^+^Ki67^+^ and glucagon^+^Ki67^+^ by 80% ± 3.6% (*P* < 0.001) and 129% ± 25% (*P* < 0.01), respectively (Figure 1, B and C). Similarly, β-alanine administration significantly increased costaining of insulin^+^Ki67^+^ and glucagon^+^Ki67^+^ by 123% ± 19% (*P* < 0.001) and 157% ± 29% (*P* < 0.01), respectively (Figure 1, B and C). These observations suggest glycine and β-alanine induce proliferation of both β and α cells in mouse islets.

**Figure 2:**
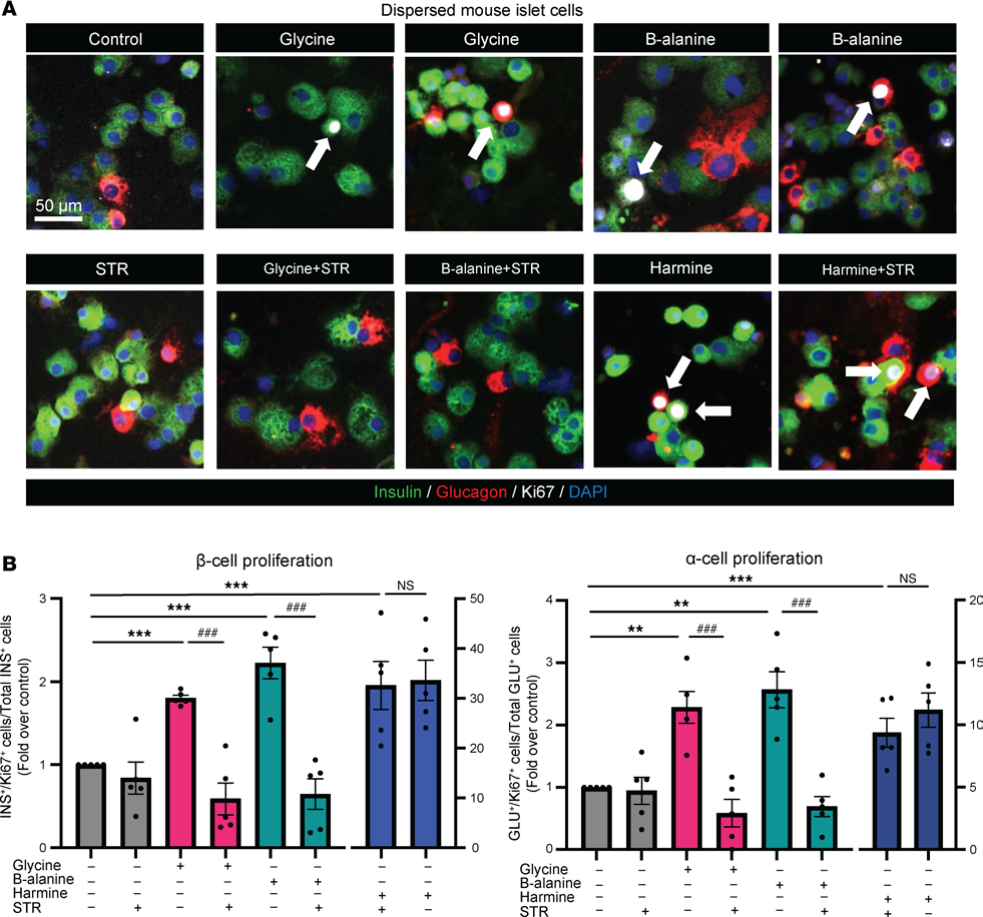
Glycine and β-alanine stimulate both β cell and α cell proliferation in mouse islets via glycine receptor in vitro. (**A** and **B**) Representative images of Ki67^+^ cells and islet cell proliferation in dispersed mouse islets treated with vehicle, 1 mM glycine, 1 mM β-alanine, or 10 μM Harmine, with or without 1 μM strychnine for 5 days (*n* = 5 mice used for each treatment). Data are depicted as mean ± SEM. Statistical significance was determined by using unpaired *t* test, with Holm-Bonferroni correction applied for multiple comparisons. ***P < 0.001, **P < 0.01, compared with the control group. ^###^*P* < 0.001, compared with the glycine or β-alanine treatment group. GlyR, glycine receptor; STR, strychnine. Scale bar: 50 μm. White arrows indicate Ki67^+^ proliferating α cells and β cells.

There are 4 known isoforms of α-subunit (α1–α4) and a single β-subunit of GlyR in most species (18, 19) including humans, where *GLRA4*, a gene encoding GlyRα4, is thought to be a pseudogene (28). In line with previous studies (29–32), we demonstrated that GlyRs were expressed in mouse and human islets by performing quantitative PCR (qPCR) (Supplemental Table 1; supplemental material available online with this article; https://doi.org/10.1172/jci.insight.178754DS1) and immunofluorescence staining (Supplemental Figure 1). Next, strychnine, a specific GlyR inhibitor (19), was used to determine whether glycine/β-alanine–induced islet proliferation was mediated through the activation of GlyR. With strychnine, the proliferation of both β cell and α cell induced by glycine and β-alanine was blocked (*P* < 0.001) (Figure 2, A and B). In contrast, harmine-induced proliferation remained unaffected by strychnine (Figure 2, A and B). This suggested that, unlike harmine action, glycine and β-alanine induced the islet cell proliferation through GlyR.

**Figure 3:**
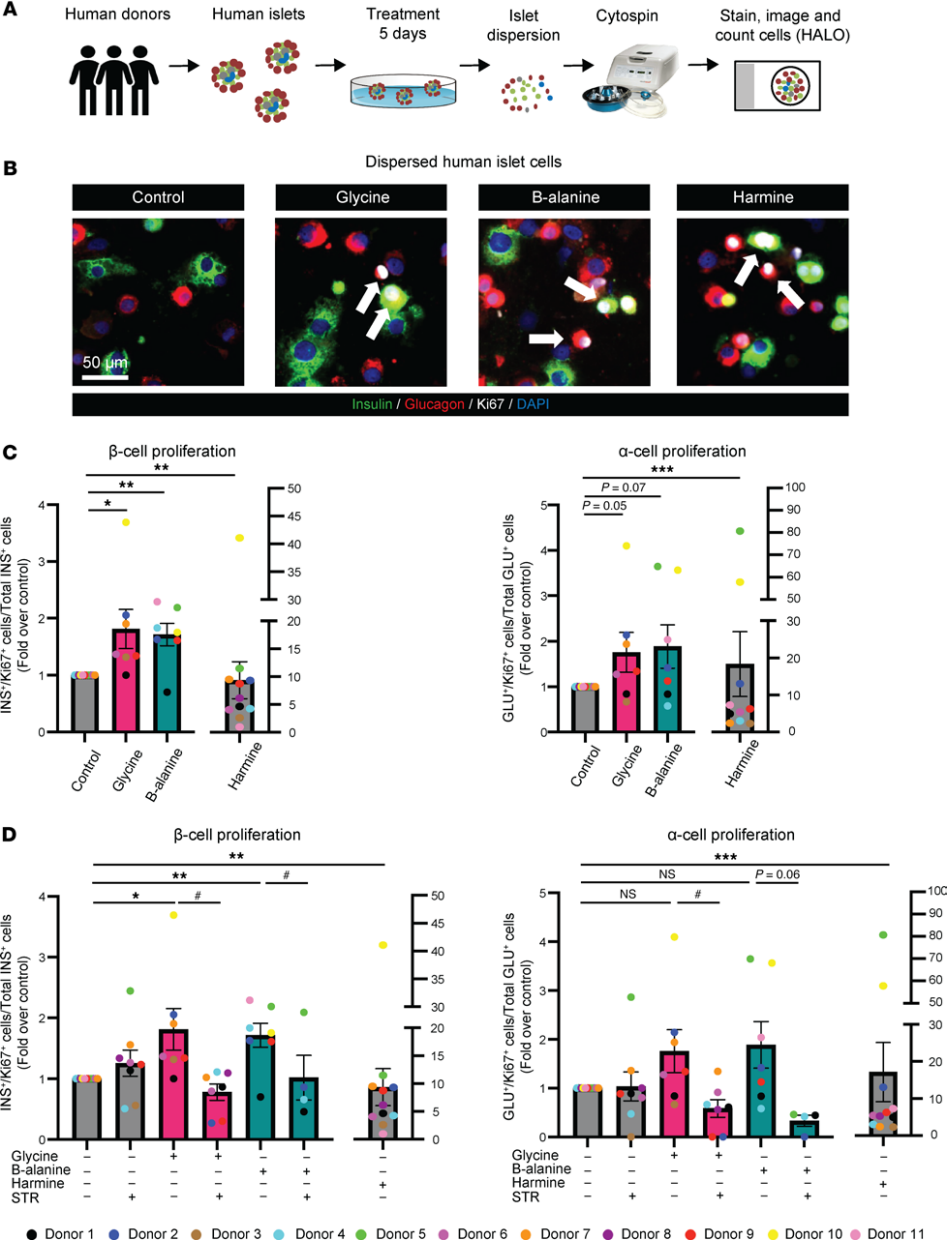
Glycine and β-alanine stimulate both β cell and α cell proliferation in human islets via glycine receptor in vitro. (**A**) Workflow for in vitro treatment of human islets. (**B** and **C**) Representative images of Ki67^+^ cells and islet cell proliferation in human islets treated with vehicle, 1mM glycine, or 1 mM β-alanine for 5 days in vitro. Harmine (10 μM) was used as a positive control for islet cell proliferation. We used 30 islets per donor, with an average of 7,490 cells per sample to derive the data. Cell proliferation was calculated by normalizing Ki67^+^ β cell/α cell numbers to total β cell/α cell numbers on cytospin slides. Scale bar: 50 μm. White arrows indicate Ki67^+^ proliferating α cells and β cells. (**D**) Islet cell proliferative in primary human islets treated with vehicle, 1 mM glycine, or 1 mM β-alanine, with or without 1 μM strychnine for 5 days. Data are depicted as mean ± SEM. Statistical significance was determined by using Mann-Whitney *U* test or unpaired *t* test dependent on dataset normality test, with Holm-Bonferroni correction applied for multiple comparisons. *P < 0.05, **P < 0.01, ***P < 0.001, compared with the control group. ^#^*P* < 0.05, compared with the glycine or β-alanine treatment group. STR, strychnine. We used islets from 11 donors, 7 of 11 donors have accessible HbA1c values, all falling within the normal range.

In cultured human islets, 5 days treatment of glycine or β-alanine enhanced the proliferation of both β and α cells, compared with corresponding controls (Figure 3, A–C). Glycine treatment significantly increased costaining of insulin^+^Ki67^+^ or glucagon^+^Ki67^+^ by 81% ± 34% (*P* < 0.05) or 76% ± 44% (*P* = 0.05), respectively (Figure 3, B and C). Similarly, β-alanine administration also increased costaining of insulin^+^Ki67^+^ or glucagon^+^Ki67^+^ by 72% ± 20% (*P* < 0.01) or 89% ± 48% (*P* = 0.07), respectively (Figure 3, B and C). Like the observations in mouse islets, we found that these positive effects on cell proliferation induced by glycine/β-alanine could be blocked by cotreatment with strychnine (Figure 3D), implicating GlyR activation in primary human islets.

### Effects of glycine or β-alanine treatment on insulin secretion and content in mouse islets in vitro.

**Figure 4:**
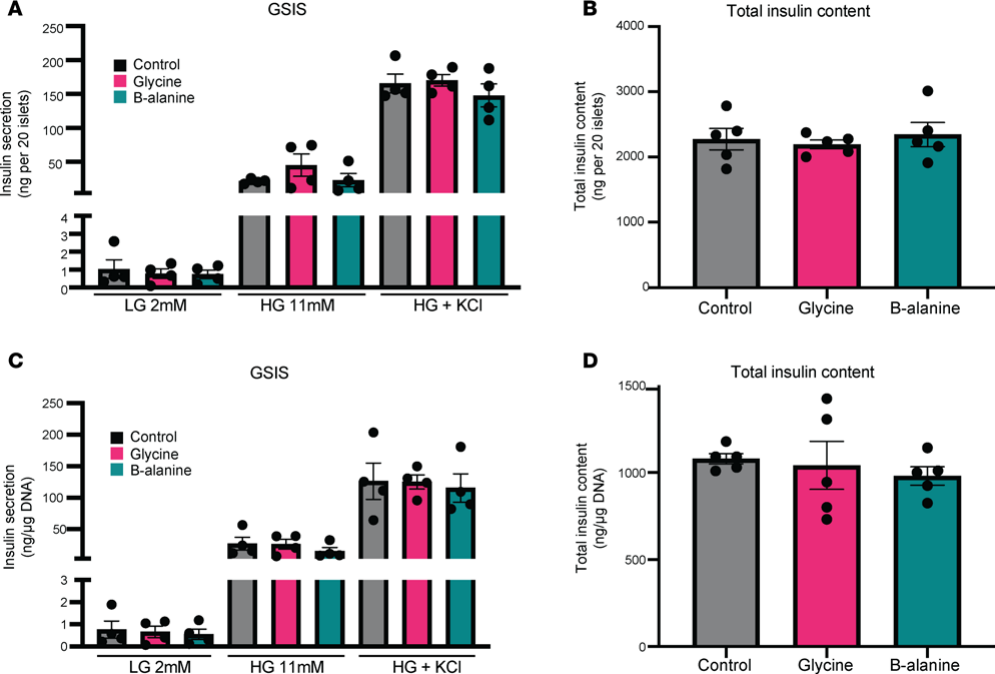
Glycine and β-alanine treatment had no effect on glucose-stimulated insulin secretion (GSIS) and total islet insulin content in mouse islets in vitro. (**A**) Mouse islets were treated with vehicle, 1 mM glycine or 1 mM β-alanine for 5 days in vitro. Insulin secretion under the treatment of low glucose (2 mM), high glucose (11 mM), and high glucose + KCl was measured. (**B**) Total insulin content of 20 islets treated with vehicle, 1 mM glycine, or 1 mM β-alanine for 5 days in vitro. (**C** and **D**) Insulin secretion and total insulin content normalized to total DNA content. Data are depicted as mean ± SEM. *n* = 4–5 mice used for each treatment. Statistical significance was determined by using unpaired *t* test, with Holm-Bonferroni correction applied for multiple comparisons. There were no significant differences among the groups, with *P* > 0.05.

After treating isolated mouse islets with glycine or β-alanine for 5 days, we evaluated glucose-stimulated insulin secretion (GSIS). No change was observed in insulin secretion under the treatment of low glucose (2 mM), high glucose (11 mM), or high glucose + KCl, the latter of which depolarizes β cells to maximally stimulate insulin exocytosis (Figure 4A). Moreover, total insulin content was not changed (Figure 4B). After normalization to DNA content/cell number, there was no significant difference in insulin secretion or total insulin content among the control, glycine, and β-alanine group (Figure 4, C and D). In an acute study on human islets where glycine was added during GSIS, it was found that the addition of glycine did not significantly affect insulin secretion, regardless of treatment (low glucose [2 mM] or high glucose [11 mM]; Supplemental Figure 2). Moreover, neither glycine nor β-alanine induced glucagon secretion in vitro in mouse islets, with l-arginine serving as a positive control (Supplemental Figure 3).

### Islet proliferation and expansion is enhanced in mice supplemented with glycine and β-alanine, but no changes are observed in glucose tolerance.

**Figure 5:**
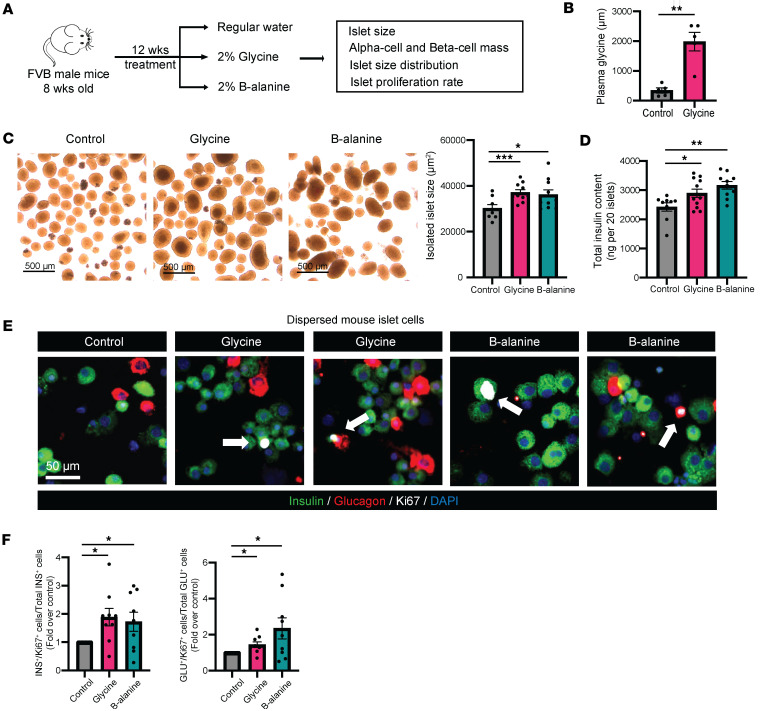
Glycine and β-alanine stimulate both β cell and α cell proliferation in mouse islets in vivo. (**A**) Workflow for in vivo oral administration of 2% glycine and 2% β-alanine drinking water to mice and subsequent assessment. (**B**) Plasma glycine concentration. (**C**) Isolated islet size. Scale bars: 500 μm. (**D**) Total insulin content in 20 isolated mouse islets. (**E** and **F**) Representative images of Ki67^+^ cells and islet cell (both β cell and α cell) proliferation in mice treated with vehicle, 2% glycine, or 2% β-alanine for 12 weeks in vivo. We used 30 islets per mouse, with an average of 17,778 cells per sample to derive the data. Data are depicted as mean ± SEM. *n* = 7–9 in each group. Scale bar: 50 μm. White arrows indicate Ki67^+^ proliferating α cells and β cells. Statistical significance was determined by using unpaired *t* test, with Holm-Bonferroni correction applied for multiple comparisons where appropriate. *P < 0.05, **P < 0.01, ***P < 0.001, compared with the control group.

To explore the effects of glycine and β-alanine administration on islet cell proliferation in vivo, we used wild-type FVB strain (WT FVB) male mice supplemented with 2% glycine or 2% β-alanine in drinking water for 12 weeks and performed IHC to evaluate β cell and α cell proliferation (Figure 5A). In control mice, without glycine or β-alanine supplement, we found that the circulating glycine was 344.6 ± 84.9 μM in the plasma (Figure 5B), which is consistent with previous reports (5). After oral glycine treatment, there was a 5.7-fold increase in circulating glycine concentration (1,981.2 ± 314.2 μM versus 344.6 ± 84.9 μM in the control) (Figure 5B). Targeted metabolomics was used to assess circulating amino acid levels after glycine treatment, revealing that, aside from isoleucine, the levels of 19 other amino acids remained unchanged following glycine administration (Supplemental Figure 4).

**Figure 6:**
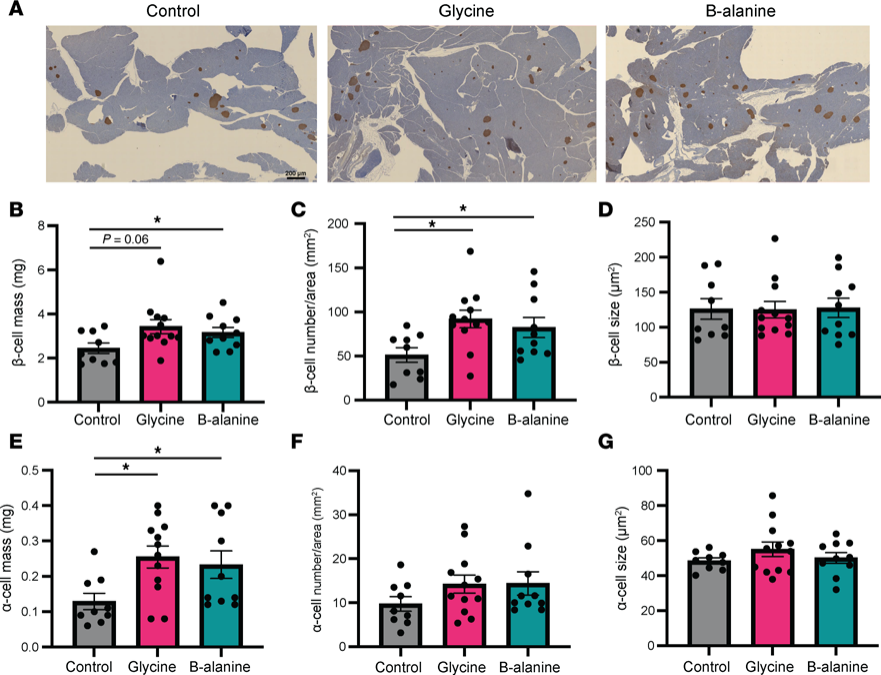
Glycine and β-alanine stimulate both β cell and α cell proliferation in mouse islets in vivo. (**A**) Representative images of insulin-stained pancreatic sections from mice administered glycine or β-alanine for 12 weeks. Scale bar: 200 μm. (**B**–**G**) Analysis of these sections were performed by calculating β cell mass (**B**), β cell number/area (**C**), average β cell size (**D**), α cell mass (**E**), α cell number/area (**F**), and average α cell size (**G**). *n* = 9–12 samples were analyzed in each group, with sections from 2 different levels in each sample stained and analyzed. Statistical significance was determined by using unpaired *t* test (**B**, **C**, **E**, and **G**) or Mann-Whitney *U* test (**D** and **F**) dependent on dataset normality test, with Holm-Bonferroni correction applied for multiple comparisons. *P < 0.05, compared with the control group.

Examining isolated islets, we observed that the mean size of islets treated by glycine and β-alanine was significantly greater than that of the control group (Figure 5C), as was total insulin content (Figure 5D), compared with control mice. Employing Ki67 staining on dispersed islet cells, we found Ki67^+^ β cells and Ki67^+^ α cells were significantly increased in glycine and β-alanine treated groups, compared with controls (Figure 5, E and F). In further studies examining pancreatic islets, we observed a significant increase in the β cell mass, β cell number/area, and α cell mass, but no changes in the mean β or α cell size were observed (Figure 6, A–G), ruling out cell hypertrophy as a factor. These data suggest that there was a significant increase in islet cell proliferation after the administration of glycine or β-alanine.

**Figure 7:**
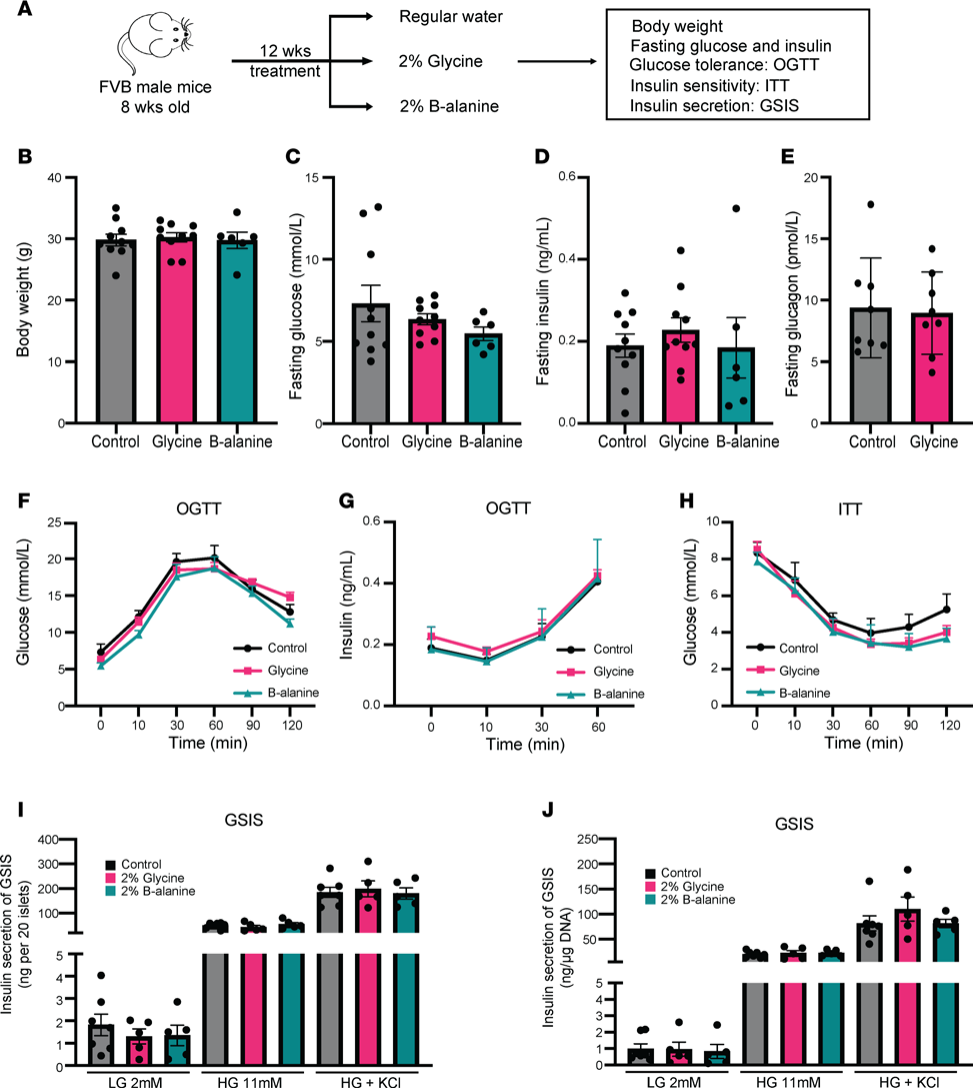
Glycine and β-alanine administration did not influence glucose homeostasis in vivo upon glucose stimulation. (**A**) Workflow for treatment and in vivo and in vitro assessment. (**B**–**H**) Mice were treated with 2% glycine or 2% β-alanine through drinking water for 12 weeks and were then assessed for glucose homeostasis by evaluating body weight (**B**), fasting glucose (**C**), fasting insulin (**D**), fasting glucagon level (mice were treated with 2% glycine or normal drinking water for 5 weeks before glucagon assessment) (**E**), glucose changes during OGTT (**F**), insulin levels during OGTT (**G**), and glucose levels during ITT (**H**). *n* = 6–10 in each group. (**I** and **J**) glucose-stimulated insulin secretion in 20 islets isolated from mice treated with vehicle, 2% glycine or 2% β-alanine, with or without normalization by DNA content/cell number. *n* = 5–7 in each group. Data are depicted as mean ± SEM. Statistical significance was determined by using unpaired *t* test (**B**, **D**, **E**, and **I**), 1-way ANOVA (**F**–**H**) or Mann-Whitney *U* test (**C** and **J**) dependent on dataset normality test, with Holm-Bonferroni correction applied for multiple comparisons.

Next, we examined the long-term effects of glycine and β-alanine on key metabolic parameters and islet function (Figure 7A). No changes were observed in body weight, fasting plasma glucose, fasting insulin, or fasting glucagon in glycine- or β-alanine–treated groups compared with the control (Figure 7, B–E). Moreover, there was no significant difference in glucose tolerance and insulin sensitivity between glycine- and β-alanine–treated groups and the controls (Figure 7, F–H). After 12 weeks of treatment, no changes in insulin secretion were observed ex vivo in the mice supplemented with glycine/β-alanine or regular water (Figure 7, I and J). These results suggested glycine or β-alanine supplementation has no effect on glucose homeostasis or insulin secretion in vivo under normal/physiological conditions.

### Treatment of glycine or β-alanine improves glucose homeostasis in mice with acute severe insulin resistance.

**Figure 8:**
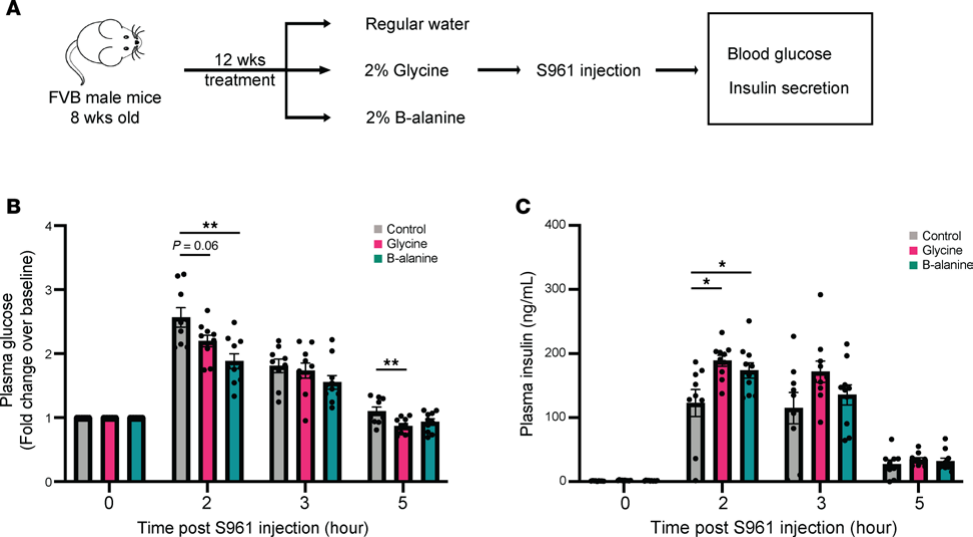
Improved glucose tolerance and insulin secretion in S961-induced transient insulin resistance in glycine- and β-alanine–treated mice. (**A**) Workflow for in vivo oral administration of 2% glycine and 2% β-alanine drinking water to mice. (**B** and **C**) Blood glucose levels and plasma insulin levels were measured hourly after S961 injection (30 nmol/kg). Data are depicted as mean ± SEM. *n* = 9–12 in each group. Statistical significance was determined by using unpaired *t* test, with Holm-Bonferroni correction applied for multiple comparisons. *P < 0.05, *P < 0.01, compared with the control group.

Next, we sought to determine whether glycine or β-alanine treatment enhanced islet function under a severe acute metabolic stress condition. The compound S961, a specific insulin receptor antagonist (33), was used to elicit insulin resistance in control and glycine- or β-alanine–treated mice (Figure 8A). Prior long-term administration of glycine or β-alanine for 12 weeks led to lower plasma glucose 2 hours after S961 injection (Figure 8B). This could be attributed to the higher plasma insulin levels observed at the same time point (Figure 8C). These data reveal that both glycine and β-alanine can enhance islet proliferation and functional capacity to secrete insulin in vivo, particularly under metabolic stress conditions such as an acute insulin resistance.

### Glycine and β-alanine induce islet proliferation via the Akt/mTOR pathway in mouse islets.

**Figure 9:**
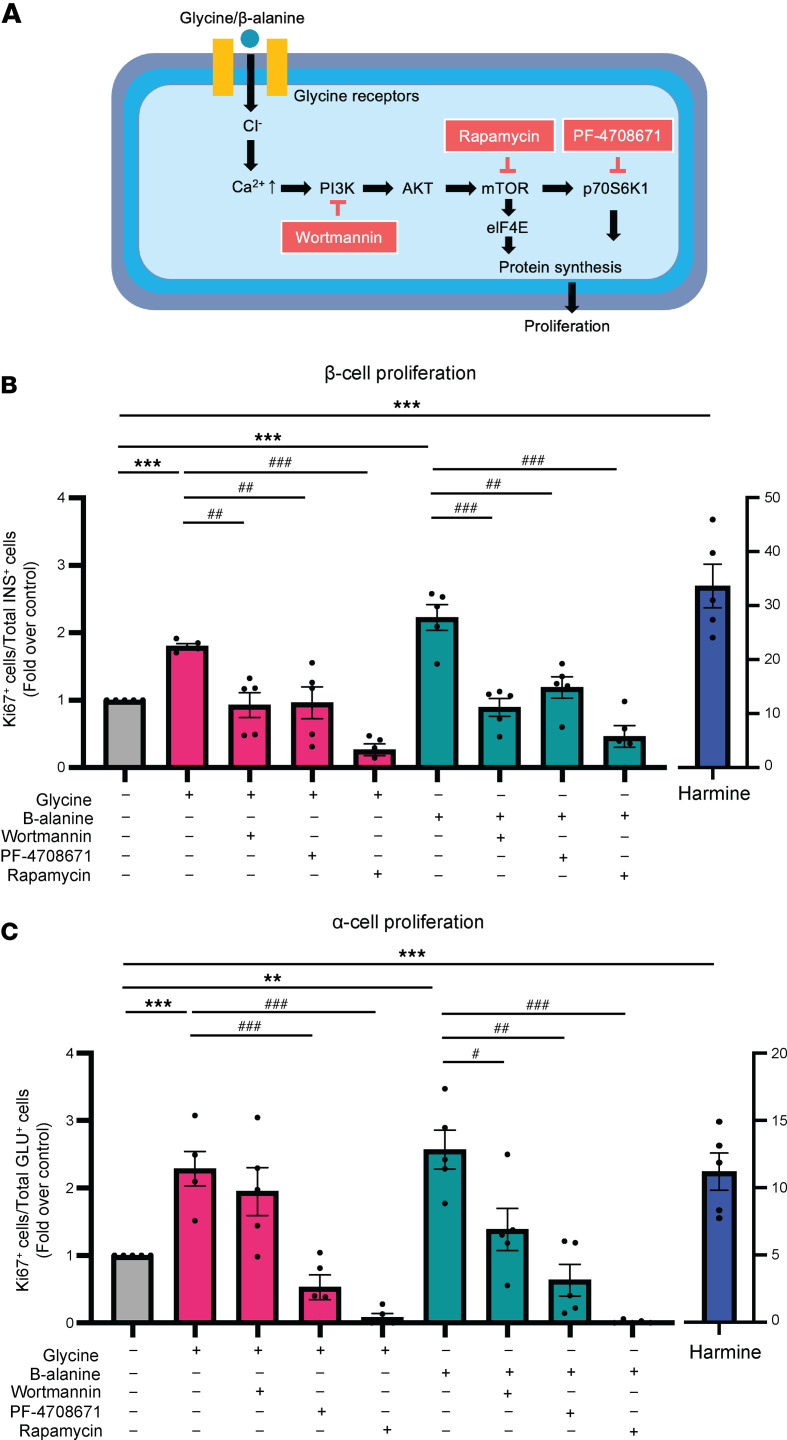
Glycine and β-alanine stimulate islet cell proliferation through PI3K/mTOR/p70S6K signaling pathway in mouse islets. (**A**) Outline of the PI3K/mTOR/p70S6K signalling pathway and selective pathway inhibitors. (**B** and **C**) Mouse islets were exposed to various treatments for 5 days in vitro (*n* = 5 for each group). Cell proliferation was assessed by normalizing Ki67^+^ β cell/α cell numbers to total β cell/α cell numbers on cytospin slides. Glycine: 1 mM; β-alanine: 1 mM; Wortmannin (PI3K antagonist): 100 nM; PF-4708671 (p70S6K inhibitor): 10 μM; Rapamycin (mTORC1/2 inhibitor): 10 nM. Data are depicted as mean ± SEM. Statistical significance was determined by using unpaired *t* test, with Holm-Bonferroni correction applied for multiple comparisons. **P < 0.01, ***P < 0.001, compared with the control group. ^#^*P* < 0.05, ^##^*P* < 0.01, ^###^*P* < 0.001, compared with the glycine or β-alanine treatment group. *n* = 4–5 in each group.

To gain mechanistic insights into how glycine or β-alanine may promote islet cell proliferation, we examined downstream pathways known to be linked to both GlyR activation and cellular proliferation (23, 26, 34). Glycine has previously been shown to activate the Akt/mTOR signaling pathway in primary mouse hepatocytes and has been linked to liver regeneration (26). Since this cytosolic signaling pathway has also been implicated in β cell growth (23), we aimed to determine if Akt/mTOR was required for glycine- and β-alanine–mediated islet cell proliferation. In isolated mouse islets, glycine or β-alanine treatment for 5 days significantly increased costaining of Ki67^+^/insulin^+^ or Ki67^+^/glucagon^+^, respectively (Figure 9, B and C), compared with the corresponding control groups. Interestingly, the addition of wortmannin (PI3K antagonist), rapamycin (mTORC1/2 inhibitor), or PF-4708671 (p70S6K inhibitor) significantly reduced the Ki67^+^/insulin^+^ or Ki67^+^/glucagon^+^ cell number, indicating glycine/β-alanine–induced islet cell proliferation effects were blunted in mouse islets (Figure 9, A–C). These data suggest that glycine and β-alanine induced islet proliferation is dependent upon an intact PI3K/mTORC1/p70S6K signaling pathway. The lack of an effect of glycine/β-alanine on insulin secretion, as shown above, would suggest that under the conditions studied, the islet proliferation effect of glycine/β-alanine is not mediated directly by insulin.

## Discussion

In our current study, we show that 5 days of treatment of glycine or β-alanine induced proliferation of pancreatic β cells and α cells in the isolated mouse islets in vitro but did not change GSIS from these islets. In vivo, in the mice supplemented with glycine or β-alanine for 12 weeks, we also observed that the proliferation of pancreatic β cells and α cells was significantly enhanced, but glucose tolerance or insulin secretion were not affected when examined under normal physiological conditions. However, when challenged by S961-induced acute insulin resistance, those mice supplemented with glycine or β-alanine had enhanced insulin secretion. Thus, the reduced glucose level may be attributed to an increased capacity to secrete insulin due to the increased β cell mass. These effects appear to require a functional PI3K/mTORC1/p70S6K signaling pathway.

Islet cells and neurons share many similarities in their physiology (35, 36), function (37–40), and gene expression (41, 42). Indeed, the inhibitory neurotransmitter GABA was detected in pancreatic β cells and was shown to play roles in insulin secretion (43) and β cell proliferation (22, 23, 44). Our previous studies demonstrated that GABA administration led to modest increases in β cell mass, insulin secretion, and glucose tolerance in vivo (22). GABA treatment also promoted β cell proliferation via the GABA_A_ receptor through the PI3K/mTORC1/p70S6K pathway in vitro (22, 23). Similar to GABA, the related inhibitory neurotransmitter glycine (45) was also detected in pancreatic β cells and, in some studies, shown to be associated with insulin secretion (5, 46). We demonstrated here that glycine can significantly enhance islet cell proliferation in both mouse and human islets, which could be explained by the fact that both GABA_A_ receptors and GlyRs are ionotropic receptors that share many physiological properties and pathways.

In neurons, glycine functions via its receptor GlyR, an ionotropic receptor exerting its effects through chloride currents (18, 19). It is composed of 4 known isoforms of the ligand-binding α-subunit (α1–α4) of GlyR (GLRA1, GLRA2, GLRA3, GLRA4) and a single β-subunit (GLRB) (18, 19) that form a pentameric structure. GlyRs are typically a heteromeric α1β receptor, consisting of 3 α1 subunits and 2 β subunits (47), or 4 α1 subunits and 1 β subunit (18). The β subunit determines the synaptic localization of the receptor and the pharmacological profile of glycinergic currents (48). Here we detected the expression of the GlyR in pancreatic β cells from both mice and humans (Supplemental Figure 1) providing molecular evidence of GlyR-mediated action of glycine. In addition to glycine, some other amino acids, including β-alanine and taurine, can also activate GlyR (19, 49). Owing to their less efficacious channel-opening equilibrium compared with glycine, β-alanine and taurine are considered as GlyR partial agonists in some studies (50). In our current study, we found that the treatment of β-alanine could stimulate the proliferation of pancreatic β cells both in vivo and in vitro. Importantly, GlyR inhibitor strychnine completely abolished the β cell proliferation induced by glycine or β-alanine. These findings suggest the stimulation of β cell proliferation by glycine or β-alanine is mediated by the activation of GlyR. Harmine, a DYRK1A inhibitor, unlike glycine, did not stimulate islet cell proliferation through GlyR, since the GlyR inhibitor strychnine could not block the proliferative effects of harmine on β and α cells. On another note, GlyR was previously found to colocalize with the GABA_A_ receptor in some hippocampal neurons (51). We do not know whether this occurs in islet cells. It would be interesting to speculate that GABA might act partially through GlyR, and glycine might act partially through GABA_A_, on the premise that the GlyR- and GABAR-induced proliferation pathways overlap. Interestingly, in addition to the β cell, we also observed that glycine could stimulate the proliferation of α cells. Since GlyR expression was also detected in the α cells of mice and humans (Supplemental Figure 1), the effects of glycine supplementation on the α cell are noteworthy. In fact, augmented α cell function may offset effects linked to increases in β cell mass on glucose tolerance. Although glycine acts as a NMDA (N-methyl-D-aspartate) receptor (NMDAR) agonist, the opening of NMDAR conductance pore requires the binding of both glycine and glutamate (52). Additionally, we have shown that blocking GlyRs could almost completely reduce the proliferation of islet cells induced by glycine or β-alanine. This further suggests that NMDAR is not likely to contribute to the cell proliferation induced by glycine/β-alanine. It is possible that glucagon secretion stimulated by either glycine or β-alanine could exert a paracrine effect on β cells to promote proliferation, potentially mediated by GLP-1 and/or glucagon receptors, as suggested by previous studies (53–56). However, in the current study, we did not observe a significant increase in glucagon secretion with glycine or β-alanine treatment in vitro, nor was there an increase in plasma glucagon levels following 5-week glycine treatment in vivo. Additionally, in isolated human and mouse islets, the GlyR antagonist strychnine completely blocked the proliferative effects of glycine and β-alanine. This strongly indicates that their mechanism of action is mediated through the GlyR and is independent of glucagon secretion, as strychnine did not inhibit glucagon release. Nevertheless, we cannot fully rule out the involvement of glucagon from α cells in driving islet cell proliferation. It is also possible that glucagon secretion at the islet level is increased but remains below detectable levels in plasma, potentially influencing both β and α cell proliferation. Additionally, in vivo, glycine and β-alanine may influence circulating levels of GIP and GLP-1, potentially affecting insulin secretion and islet cell proliferation, which warrants further investigation.

The proliferation of pancreatic β cells is regulated by multiple pathways including Akt-mTORC1, Wnt, ERK1/2, and JAK-STAT (34). We demonstrated here that specific inhibitors of PI3K, mTORC1, and p70S6K could abolish the β cell proliferation induced by GlyR activation, suggesting the integrity of the PI3K/mTORC1/p70S6K signaling pathway is required for the glycine/β-alanine–induced cell proliferation. The PI3K signaling pathway plays roles in the regulation of β cell function with a wide-ranging network of mediators (57). Akt/PKB, a downstream target of PI3K, regulates the proliferation of β cells through modulation of its downstream genes, including forkhead box protein O1 (*FOXO1*) (58), glycogen synthase kinase 3 (*GSK3*) (59), and mTOR (60). PI3K/Akt has been shown to suppress tuberous sclerosis complex 1/2 (TSC1/2), an upstream negative regulator of mTORC1, which then acts to promote mRNA translation and synthesis of ribosomes, enhancing β cell proliferation (60, 61). Interestingly, we observed that the Akt/mTORC1/p70S6K proteins, downstream of PI3K, were important for both glycine and GABA receptor–induced β cell proliferation (22, 23), suggesting that they have common mechanisms/pathways of proliferation. However, we cannot exclude the possible involvement of other signaling pathways in addition to PI3K/Akt/mTORC1 that warrant further investigation. Additionally, studies have demonstrated a correlation between the degree of phosphorylation of the PI3K and Akt signaling pathway and calcium influx in elongated osteoblasts (62). This relationship was also observed in central nerve terminals, where localized [Ca^2+^]_i_ influx triggered the activation of PI3K through an unknown calcium sensor, leading to an increase in Akt phosphorylation (63). It remains to be determined whether similar connections exist in pancreatic β cells, and further investigation is necessary to confirm their presence.

There are of course limitations to the methods used in this paper, including that most experiments in the study relied on the use of chemical inhibitors. This raises the possibility of off-target effects. Therefore, further studies employing genetic models specific to the GlyR are warranted. In addition, this study was conducted under standard chow–diet conditions. It would be important to conduct studies involving high-fat diet feeding to strengthen the rationale for glycine supplementation to combat impaired islet function associated with T2D, for example.

### Conclusions.

In summary, we have shown that the amino acid/inhibitory neurotransmitter glycine can stimulate the proliferation of pancreatic β cells in both human and mouse islets. This effect is mediated via the activation of the GlyR and the downstream PI3K/mTORC1/p70S6K signaling pathway. Importantly, this enhanced proliferation of pancreatic β cells could result in elevated insulin secretion upon S961-induced acute insulin resistance that was associated with lower blood glucose. Further studies probing other potential signaling pathways linked to GlyR activation and their links to GABA_A_ receptors are warranted. In summary, our study provides insights into potentially employing glycine supplementation to enhance β cell function.

## Methods

### Sex as a biological variable.

In human studies, islets from both male and female donors were used as shown in Supplemental Table 2. In mouse studies, male mice were used for in vivo studies and islets from male mice were used in in vitro studies. Female mice experience cyclical hormonal changes due to their estrous cycle, which can influence metabolism and glucose homeostasis, introducing additional complexity and potential confounders in experiments to assess islet function. It is unknown whether the findings are relevant to female mice.

### Animals.

Eight-week-old FVB male mice were obtained from Charles River Laboratories. Mice were kept at 22°C, on a 12-hour light/12-hour dark cycle with free access to normal chow diet. Mice were administered 2% glycine or 2% β-alanine (MilliporeSigma) via drinking water for 12 weeks, and the quantity of fluid consumed was not controlled. Age-matched male FVB mice with access to normal drinking water served as controls.

### Mouse and human islets.

Mouse islets were isolated as described previously (22, 23). Briefly, mice were anesthetized by isoflurane and then sacrificed by cervical dislocation. The pancreas was surgically isolated and perfused through the bile duct and digested by 3 mL of 0.8 mg/mL Collagenase V (MilliporeSigma) (dissolved in 1640 RPMI, MilliporeSigma). The perfused pancreas was then excised and placed in 5 mL of 0.8 mg/mL Collagenase V solution at 37°C for 11 minutes. The digestion was stopped by the addition of 1640 RPMI supplemented with 10% FBS (MilliporeSigma) and 1% penicillin/streptomycin (MilliporeSigma). Islets were hand picked under a dissecting microscope. Human islets from healthy donors were obtained from the Alberta Islet Distribution Program (AIDP, University of Alberta, Edmonton, Alberta, Canada), the Alberta Diabetes Institute IsletCore (ADI IsletCore, University of Alberta, Edmonton, Alberta, Canada) and the University Health Network Human Islet Isolation Program (UHN, Toronto, Ontario, Canada). Human islets were hand picked and cultured in a low-glucose DMEM (Gibco) that was supplemented with 10% FBS and 1% penicillin/streptomycin before experiments. Since interstitial glycine concentrations are expected to exceed the circulating level (~300 μM), in particular because pancreatic β cells store and release glycine as previously described (5), we rationalized the use of higher concentration (1 mM) to treat primary mouse and human islets in vitro in the present study. Furthermore, the concentrations of glycine and β-alanine required for generating a half-maximal response (EC_50_) of membrane currents were similar at both α1 and α2 subunit–containing GlyRs (50). Therefore, intact FVB mouse or human islets were treated with 1 mM glycine, 1 mM β-alanine (3-amino propanoic acid), with or without PI3K/AKT/mTOR signaling pathway inhibitors (30 nM PF-4708671, 10 μM rapamycin, 10 nM wortmannin) for 5 days before further experiments. Harmine, a tyrosine-phosphorylation-regulated kinase 1A (DYRK1A) inhibitor and mitogenic compound, was used as positive control of islet cell proliferation (27). All pharmacological agents were obtained from MilliporeSigma.

### GSIS.

GSIS was performed to assess insulin secretion in response to glucose as previously described (64, 65). Briefly, intact islets were picked into KRB buffer (containing 2 mM glucose) for 1.5 hours as a preincubation step. Then islets were transferred into 1.5 mL microcentrifuge tubes (20 islets per tube) and treated with low glucose (2 mM glucose), high glucose (11 mM glucose), or high glucose with KCl (11 mM glucose + 25 mM KCl) at 37°C for 1 hour. All pharmacological agents were obtained from MilliporeSigma. After 1-hour stimulation, the tubes were centrifuged for 10 minutes at 9,391*g*, and supernatant was collected for insulin assay. Secreted insulin concentrations were quantified by Homogenous Time-Resolved Fluorescence (Cisbio), and the results were normalized to total DNA content.

### Cytospin, immunofluorescence, and confocal microscopy.

Cytospin and immunofluorescence staining were performed as previously described (22, 23). Briefly, after 5 days of treatment with either glycine, β-alanine, or control, intact mouse or human islets were dissociated into single-islet cells using TrypLE (Thermo Fisher Scientific). Dispersed cells were then loaded onto slides using a Shandon Single Cytofunnel (Thermo Fisher Scientific). The slides were fixed with 4% paraformaldehyde, and then 0.2% Triton-X100 was added on to each slide, and nonspecific reactions were blocked with addition of 5% BSA. Pancreatic sections were then probed with anti-insulin (IR00261-2, Agilent Technologies), anti-glucagon (ab10988, Abcam) and anti-Ki67 (ab16667, Abcam) at 4°C overnight. Secondary anti–guinea pig Alexa Fluor 488 (ab150185, Thermo Fisher Scientific), anti-mouse Alexa Fluor 555 (ab150114, Abcam), and anti–rabbit Cy5 (A10523, Thermo Fisher Scientific) were used to detect the peptides of interest. Images were obtained using the Zeiss Axioscan Slide Scanner, and all image quantifications were carried out by HALO (Indica Labs, v.2.0.1145.14) as previously described (22, 23). For HALO analysis, DAPI and Ki67 were set as nuclear stains, whereas glucagon and insulin were set as cytoplasmic markers. A final optimized algorithm was used to analyze all annotated slides, and the performance of the algorithm was confirmed manually for each slide (22, 23).

### IHC.

To assess pancreatic islet anatomical parameters in mice, the whole pancreas was excused, weighed, and then fixed in 10% neutral buffered formalin for 4–24 hours before being embedded in paraffin. The whole pancreas was stretched in the paraffin block to maximize the total pancreatic area on each section. Sections within the middle layer, which encompasses the largest pancreatic area, were stained for insulin and glucagon as previously described (22, 23). Images were obtained using the Zeiss Axioscan Slide Scanner, and image quantifications were carried out by HALO. The insulin^+^ staining area was used to define each islet in the pancreatic section. Islet size distribution was calculated by obtaining the percentage of islet in specified size ranges out of the total islet number. The β cell mass was calculated by multiplying the average insulin^+^ area in relation to the whole pancreatic area with the pancreatic weight of the corresponding animal as we previously described (66). The β cell number of each section was normalized by the corresponding whole-pancreatic section area. The average β cell size was calculated as the insulin^+^ area divided by the number of β cells detected in each section. Two distinct sections from each animal were used for the analysis stated above.

### In vivo glucose homeostasis assessment with glucose- and insulin-tolerance tests.

After 12 weeks on the glycine or β-alanine–supplemented water or control water, all mice were fasted overnight (~14 hours), and a 2 g/kg bolus of glucose was administered by oral gavage as previously described (67, 68). Blood glucose was monitored using a Contour glucometer (Ascensia Diabetes Care) at 0, 10, 30, 60, 90, and 120 minutes. To measure plasma insulin levels during fasting and after an oral glucose-tolerance test (OGTT), tail vein blood samples were collected at 0, 10, 30, and 60 minutes during OGTT using EDTA-coated microvettes (Sarstedt). Plasma was prepared by centrifugation at 3,381*g* for 10 minutes at 4°C. Insulin levels were then analyzed by ultrasensitive mouse ELISA (80-INSMSU-E01, ALPCO). An insulin-tolerance test (ITT) was performed on mice that were fasted for 5 hours and treated with insulin (0.5 IU/kg) by i.p. injection. Tail vein blood glucose was measured at 0, 10, 30, 60, 90, and 120 minutes using a Contour glucometer.

### qPCR.

qPCR analysis was performed as previously described (69, 70). Primers were designed with the online tool Primer3web (version 4.1.0, https://primer3.ut.ee/) and are listed in Supplemental Table 1. Data were normalized to mouse or human β-actin mRNA.

### Targeted metabolomics.

The metabolomics dataset was obtained by performing targeted metabolomics as previously described (66, 71, 72). Briefly, targeted metabolomics was performed on fasting plasma samples. The Absolute IDQ p180 kit (Biocrates Life Sciences) was used to quantify the metabolites. All amino acids were analyzed using either deuterated or ^13^C stable isotope–labeled internal standard and measured by an Agilent 1290 HPLC stack connected to a SCIEX QTRAP 5500 mass spectrometer. All analyses were performed by the Analytical Facility for Bioactive Molecules (The Hospital for Sick Children) without disclosure of group allocation.

### Glucagon assessment.

Mice were fasted for 4 hours before blood was drawn for glucagon measurements. Blood was collected using heparin-coated tube with the addition of diprotin-A (I-9759, Sigma-Aldrich), EDTA (EDT001.500, BioShopa), and aprotinin-A (A6279, Sigma-Aldrich). Plasma glucagon was determined using Glucagon ELISA - 10 μL (10-1281-01, Mercodia). Isolated islets were preincubated in KRB buffer containing 2 mM glucose for 1 hour; then, islets were transferred into 1.5 mL microcentrifuge tubes (20 islets per tube) and treated with low glucose (2 mM glucose), low glucose with either 1 mM β-alanine or 1 mM glycine, and low glucose with 5 mM l-arginine at 37°C for 30 minutes. Supernatants were collected for ex vivo glucagon quantification using Ultrasensitive Glucagon ELISA (48-GLUHUU-E01, ALPCO).

### Statistics.

Data normality was assessed graphically using histograms. The unpaired 2 tailed *t* test, 1-way ANOVA, or Mann-Whitney *U* test was applied for statistical comparisons, depending on data distribution. The Holm-Bonferroni correction was used to adjust for multiple comparisons and control the type I error rate. Adjusted *P* < 0.05 were considered statistically significant.

### Study approval.

All animal experiments were approved by the University of Toronto animal care committee (no. 20011576).

### Data availability.

All data used in the paper are available. Values for all data points in graphs are reported in the Supporting Data Values file.

## Author contributions

ZZ contributed conceptualization, data analysis, methodology, original draft preparation, and review and editing. WWY contributed data analysis as well as review and editing. ALP contributed data analysis, original draft preparation, and review and editing. DSW contributed data analysis as well as review and editing. AU contributed data analysis and review. AB contributed data analysis and review. SAL and JLB contributed data analysis and review. IS contributed data analysis as well as review and editing. BAO contributed review and editing. FFD contributed conceptualization, writing, and editing. MBW contributed conceptualization, supervision, resources, funding acquisition, methodology, investigation, writing, and editing. All authors read and approved the final manuscript.

## Supplementary Material

Supplemental data

Supporting data values

## Version 1 04/22/2025

Electronic publication
