# Supplementary material for: Glycine receptor activation promotes pancreatic islet cell proliferation via the PI3K/mTORC1/p70S6K pathway
Source: JCI Insight. 2025 Apr 22;10(8):e178754. doi: 10.1172/jci.insight.178754 (PMC12016933; doi:10.1172/jci.insight.178754)
Supplement: Supplemental data [file jciinsight-10-178754-s065.pdf]

Table S1 Primer sequences used for qPCR

Gene	Forward	Reverse
B-actin (mouse)	CTGAATGGCCCAGGTCTGA	CCCTGGCTGCCTCAACAC
GLRA1 (mouse)	CTGGCCATCTCGACAGGAAA	AGCTAAGGTACCGCGGACAG
GLRA2 (mouse)	TTGACCTGACCATGATGCCC	TAGCTGCCGGTACATTCCTG
GLRA3 (mouse)	AACAAACAGTGCGCGATCTC	GTTGGGTCTGATTCTTGCATCA
GLRA4 (mouse)	GGAGAGGAAAGAGCGCTCAA	AGGAAGAGGCTTGCTGGAAC
GLRB (mouse)	ACCCCAGACTCAAGCTACCT	GACGTCTCCATCCCGAAAGA
KCNJ11 (mouse)	TCGCCCACAAGAACATTCGA	AGGAGTGGATGCTTGTGACG
B-actin (human)	GTGCTATCCCTGTACGCCTC	AGGTAGTCAGTCAGGTCCCG
GLRA1 (human)	ACAGCAATACTCTTCGCGCT	GTTAAAGGGAGGCGGGGAAC
GLRA2 (human)	AATGATTCACGGCTGGCGTA	TCCGTAGCAATTTGTTGTCAGT
GLRA3 (human)	TGGAGCGACAAATGGGATACT	GGAAGCTCGTGATCCTGAACT
GLRB (human)	CTGAATCCTGCTATGGTCACC	AGCACACTTTAAAACGAGCTGA
KCNJ11 (human)	GCTCCTCCCAACCCAATTCA	ACTTCTTGGAGCCACAGACG

Table S2: Donor Information

Donor#	Islet bank ID	Source	Sex	Age	BMI	HbA1c (%)	Islet Particle index
**Donor 1**	R330	ADI	Female	39	24.2	5.1	1.09
**Donor 2**	R338	ADI	Male	30	25.5	5.3	0.79
**Donor 3**	R340	ADI	Male	36	23.3	5.3	1.02
**Donor 4**	R341	ADI	Male	42	30.0	/	1.24
**Donor 5**	R356	ADI	Female	45	29.7	5.1	0.63
**Donor 6**	R413	ADI	Male	41	33.6	5.6	0.83
**Donor 7**	H2309	AIDP	Male	32	22.9	5.6	0.811
**Donor 8**	H2331	AIDP	Male	36	25.0	5.7	/
**Donor 9**	H2346	AIDP	Male	59	29.5	5.9	/
**Donor 10**	H2366	AIDP	Male	60	23.8	5.6	/
**Donor 11**	UHN210616	UHN	Female	39	34.8	/	/
**Donor 12**	R326	ADI	Male	26	27	5.5	1.52

ADI, Alberta Diabetes Institute IsletCore; AIDP, Alberta Islet Distribution Program; UHN, University Health Network.

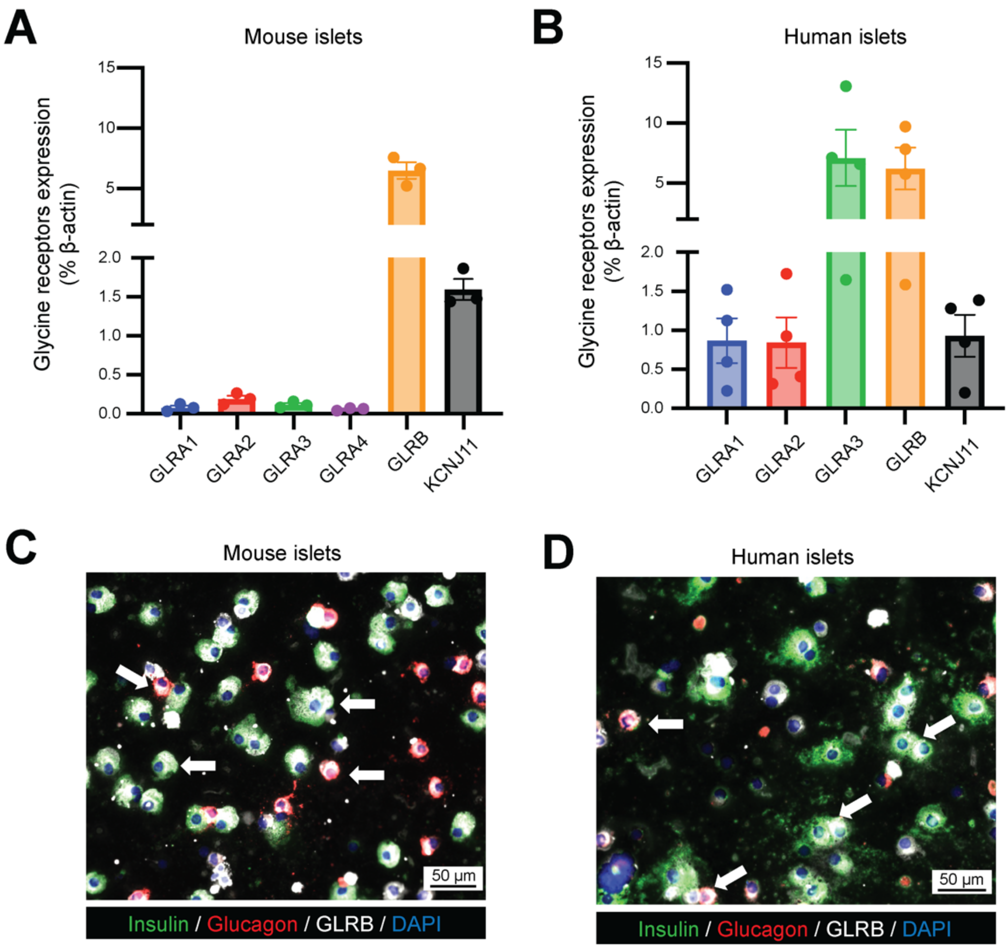

Figure S1. Glycine receptor expression in primary mouse and human islets. A) Mouse and B) human islet qPCR of glycine receptor subunits (Mouse: *GLRA1-4* and *GLRB*; Human: *GLR1-3* and *GLRB*). *KCNJ11*, a gene encoding Kir6.2 subunit of the ATP-sensitive potassium channel in the pancreatic β-cell, was used as positive control. C) and D) Representative images of GLRB positive cells in dispersed mouse and human islets. Data are depicted as mean ± SEM.

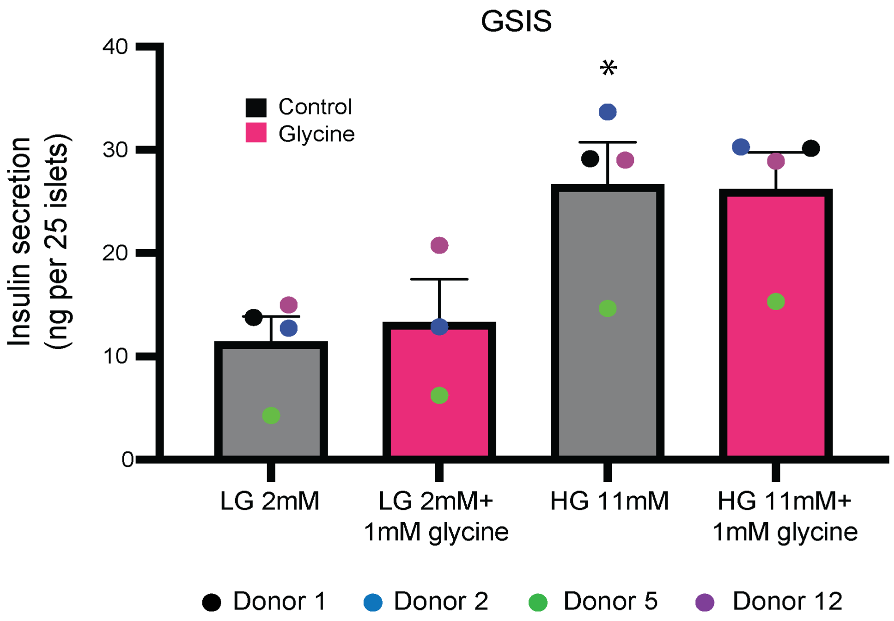

Figure S2. Glycine had no effect on glucose-stimulated insulin secretion (GSIS) in human islets. Vehicle or 1mM glycine was added during a GSIS assay performed on human islets. Insulin secretion under the treatment of low glucose (2mM), high glucose (11mM) with or without 1mM glycine was measured. Data are depicted as mean ± SEM. Statistical significance was determined by using unpaired t-test, with Holm-Bonferroni correction applied for multiple comparisons. *p-value<0.05,compared to the LG control group. LG, low glucose; HG, high glucose.

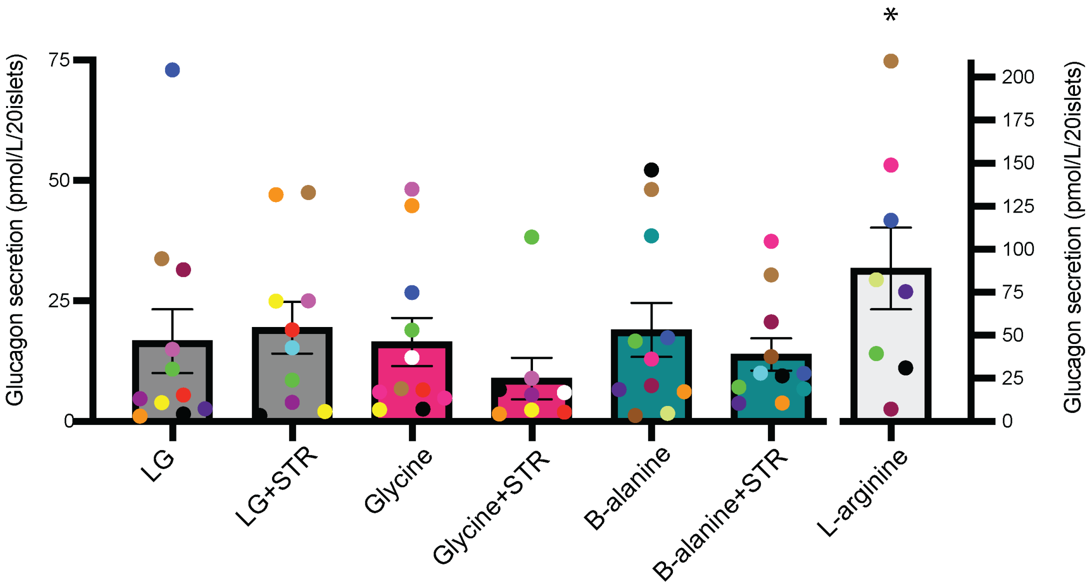

Figure S3. Glycine and β-alanine had no effect on mouse islet glucagon secretion *in vitro*. Mouse islets were treated in low glucose condition (2mM) with 1 mM glycine or 1 mM β-alanine, in the presence or absence of strychnine. A 5 mM L-arginine treatment served as a positive control. Data are expressed as mean ± SEM, with N = 8–12 per group. Each solid-colored circle represents the same islet preparation under different treatment conditions.

Statistical significance was determined by using Mann-Whitney test or unpaired t-test dependent on dataset normality test, with Holm-Bonferroni correction applied for multiple comparisons. *p-value<005,compared to the LG control group. LG, low glucose; STR, strychnine.

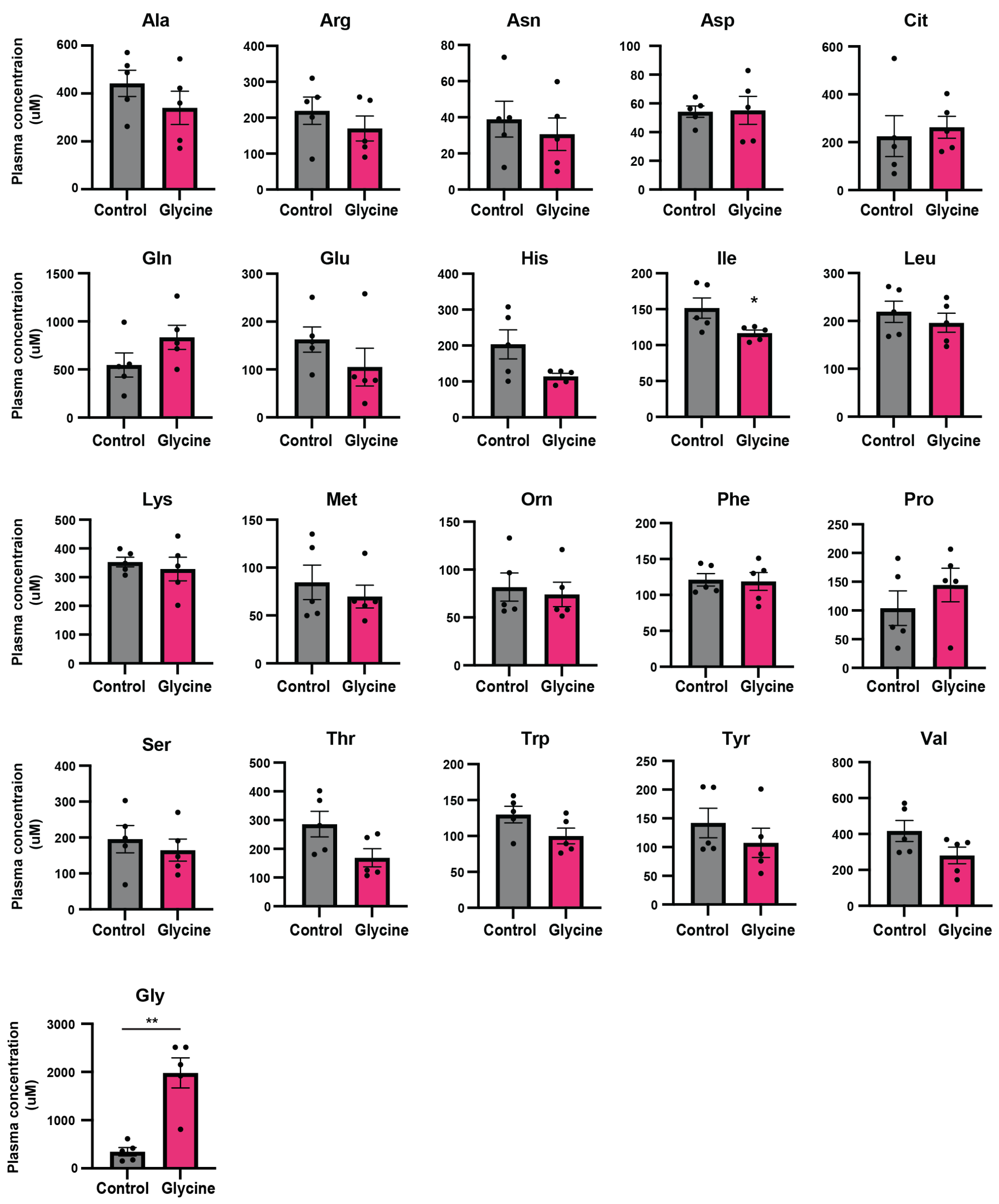

Figure S4. Glycine administration had no effect on other circulating amino acid levels *in vivo*. Mice were treated with 2% glycine or normal drinking water for 12 weeks and circulating amino acid levels were assessed using targeted metabolomics. Data are presented as mean ± SEM, with N=5 per group. Statistical significance was determined by using Mann-Whitney test or unpaired t-test dependent on dataset normality test. *P<0.05, **P<0.01, compared to the control group.
